# Immunomodulatory and Anti-inflammatory Activity *in Vitro* and *in Vivo* of a Novel Antimicrobial Candidate*

**DOI:** 10.1074/jbc.M116.750257

**Published:** 2016-10-07

**Authors:** Jlenia Brunetti, Giulia Roscia, Ilaria Lampronti, Roberto Gambari, Leila Quercini, Chiara Falciani, Luisa Bracci, Alessandro Pini

**Affiliations:** From the ‡ Department of Medical Biotechnology, University of Siena, via Aldo Moro 2, 53100 Siena, Italy,; the § Department of Life Sciences and Biotechnology, University of Ferrara, via Fossato di Mortara 74, 44121 Ferrara, Italy, and; ¶ SetLance srl, via Fiorentina 1, 53100 Siena, Italy

**Keywords:** antimicrobial peptide (AMP), drug discovery, infectious disease, inflammation, LPS, anti-inflammatory agents

## Abstract

The synthetic antimicrobial peptide SET-M33 has strong activity against bacterial infections caused by Gram-negative bacteria. It is currently in preclinical development as a new drug to treat lung infections caused by Gram-negative bacteria. Here we report its strong anti-inflammatory activity in terms of reduced expression of a number of cytokines, enzymes, and signal transduction factors involved in inflammation triggered by LPS from *Pseudomonas aeruginosa*, *Klebsiella pneumoniae*, and *Escherichia coli*. Sixteen cytokines and other major agents involved in inflammation were analyzed in macrophages and bronchial cells after stimulation with LPS and incubation with SET-M33. The bronchial cells were obtained from a cystic fibrosis patient. A number of these proteins showed up to 100% reduction in expression as measured by RT-PCR, Western blotting, or Luminex technology. LPS neutralization was also demonstrated *in vivo* by challenging bronchoalveolar lavage of SET-M33-treated mice with LPS, which led to a sharp reduction in TNF-α with respect to non-SET-M33-treated animals. We also describe a strong activity of SET-M33 in stimulating cell migration of keratinocytes in wound healing experiments *in vitro*, demonstrating a powerful immunomodulatory action generally characteristic of molecules taking part in innate immunity.

## Introduction

Antimicrobial peptides (AMPs),^3^ also known as host defense peptides, are new breakthrough molecules for the treatment of bacterial infections (1, 2). Several AMPs of natural and synthetic origin are currently being developed preclinically or clinically as new therapeutic agents for systemic, lung, and wound infections (3, 4). The growing interest in such molecules is due to the increase in bacterial multidrug resistance to traditional antibiotics and associated expectations of imminent increased medical need. Contrary to traditional antibiotics, which only kill bacterial cells or inhibit their growth, AMPs show a double mechanism that includes strong antibacterial activity with potent killing methods involving rapid membrane disruption (5) and immunomodulatory properties via activation of pro- and anti-inflammatory responses, chemoattraction, cell differentiation, activation of innate and adaptive compartments, wound healing, and apoptosis (6). All of these activities render AMPs simultaneously antibacterial and anti-inflammatory. Lung infections such as those encountered in cystic fibrosis (CF) patients, where bacteria and inflammation triggered by the infection play a major role in pathogenesis, are ideal targets for such molecules.

We isolated, optimized, and synthesized a peptide (SET-M33) that shows strong antimicrobial activity *in vitro* and *in vivo* against major Gram-negative pathogens (7–10). SET-M33 was synthesized in a branched form (11) for better stability in biological fluids (12–16). Its mode of action is based on a two-step mechanism: high affinity binding to LPS (8) and disruption of bacterial membranes. We characterized SET-M33 biological activity against a number of Gram-negative multidrug-resistant clinical isolates, including many CF isolates (9), as well as its interactions with membranes, LPS, and DNA; its *in vitro* toxicity against several eukaryotic cell lines (17); and its hemolytic activity, lack of immunogenicity (8), and efficacy in eradicating biofilms (18). SET-M33 production includes a procedure for the elimination of by-products suitable for industrial development of a peptide-based drug (17).

Preclinical development of SET-M33, currently underway, has already provided information about its therapeutic power *in vivo* in cases of sepsis, models of skin and lung infections, gene toxicity, acute toxicity *in vivo*, its propensity to select bacterial resistances, pharmacokinetics, and biodistribution (10). Because SET-M33 is a drug candidate for lung infections in CF, its potential as an anti-inflammatory agent is also of great interest. Here we analyzed its capacity to neutralize LPS, thus reducing gene and protein expression of the principal cytokines involved in inflammation triggered by LPS from *Pseudomonas aeruginosa*, *Klebsiella pneumoniae*, and *Escherichia coli*, major pathogens of lung infections, including those afflicting CF patients. Sixteen cytokines and other major factors involved in inflammation caused by bacterial LPS were analyzed in murine macrophages and human bronchial cells from a CF patient. TNF-α expression was also evaluated *in vivo* after nebulization of LPS and SET-M33 in mouse lungs. Because bacterial infections are the most significant complication encountered in wound management (19), we also evaluated the performance of SET-M33 in stimulating cell migration of keratinocytes in wound healing experiments.

## Results

### 

#### 

##### Gene Expression of Pro-inflammatory Factors in Macrophages

**FIGURE 1.**
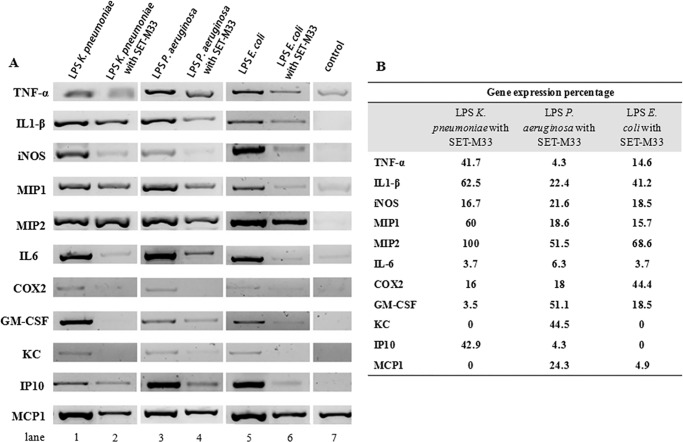
**Gene expression of pro-inflammatory cytokines and enzymes analyzed by RT-PCR.** *A*, cDNA bands indicating gene expression in cells stimulated with LPS from *K. pneumoniae*, *P. aeruginosa*, or *E. coli* (*lanes 1*, *3*, and *5*, respectively) or in cells stimulated with LPS in the presence of SET-M33 (*lanes 2*, *4*, and *6*, respectively). *Control* (*lane 7*) indicates the basal level of gene expression in unstimulated cells. *B*, percentage of gene expression after LPS stimulation and SET-M33 treatment. The value 100% corresponds to gene expression in LPS-stimulated cells after subtraction of the basal level. Densitometric analysis was carried out using ImageJ software. RAW264.7 murine macrophages were incubated with SET-M33 (1 μm) when stimulated with LPS from *E. coli* to study inhibition of TNF-α, IL1-β, MIP1, and MIP2; when stimulated with LPS from all bacteria to study inhibition of IL6 and COX2; and when stimulated with LPS from *K. pneumoniae* and *E. coli* to study inhibition of GM-CSF, KC, IP10, and MCP1. In all other cases, SET-M33 was used at 10 μm.

SET-M33 was tested for LPS neutralization in macrophages and, consequently, for its inhibitory effects on the inflammatory cytokines TNF-α, IL1-β, MIP1, MIP2, IL6, GM-CSF, KC, IP10, and MCP-1, on iNOS, a key enzyme in the generation of nitric oxide, and on COX-2, the enzyme responsible for formation of prostanoids. Gene expression analysis by RT-PCR showed that stimulation of RAW264.7 murine macrophages with LPS from *K. pneumoniae*, *P. aeruginosa*, or *E. coli* induced an increase in gene expression of all proteins tested. In cells stimulated with LPS at appropriate concentrations (20 ng/ml LPS from *K. pneumoniae* and *E. coli*, 50 ng/ml LPS from *P. aeruginosa*, and 100 ng/ml LPS from *E. coli* for IL1-β) and then treated with SET-M33 (1 or 10 μm as indicated in Fig. 1), expression of pro-inflammatory cytokines and enzymes was strongly inhibited (Fig. 1*A*), with the exception of MIP2 induced by LPS from *K. pneumoniae*. Fig. 1*B* shows the gene expression percentage after treatment with SET-M33 with respect to the signal obtained from untreated LPS-stimulated cells minus the basal level. SET-M33 inhibited gene expression as follows: 100% for KC and MCP1 in cells stimulated with LPS from *K. pneumoniae* and for KC and IP10 in cells stimulated with LPS from *E. coli*; >90% for IL6 in cells stimulated with LPS from all bacteria, for GM-CSF in cells incubated with LPS from *K. pneumoniae*, for IP10 in cells stimulated with LPS from *P. aeruginosa*, and for MPC1 in cells incubated with LPS from *E. coli*; >75% for iNOS and COX2 in cells stimulated with LPS from *K. pneumoniae*, for IL1-β, iNOS, MIP1, COX2, and MCP1 in cells stimulated with LPS from *P. aeruginosa*, and for TNF-α, iNOS, MIP1, and GM-CSF in cells stimulated with LPS from *E. coli*; and >30% for the other cytokines, with the exception of MIP2 in *K. pneumoniae* LPS-stimulated cells, which was not inhibited at all.

##### NF-κB Inhibition

**FIGURE 2.**
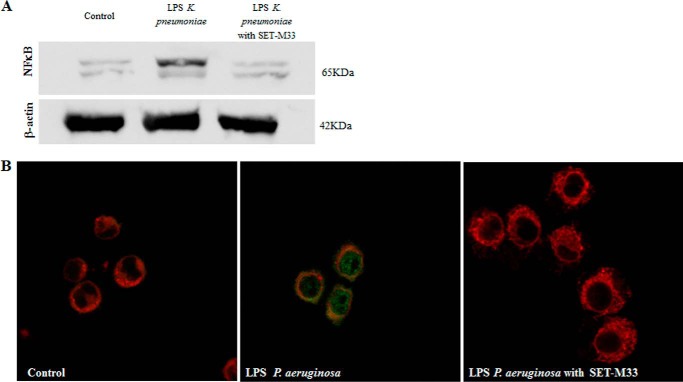
**NF-κB inhibition in macrophages.** *A*, Western blotting showing protein expression in cells stimulated with 25 ng/ml LPS from *K. pneumoniae* (*center*) and treated with 10 μm SET-M33 (*right*). The control is the basal level in cells not stimulated with LPS (*left*). The peptide restored the NF-κB signal as in the control band. The presence of two bands is due to the heterodimeric form of NF-κB. β-actin was used as an experimental control (*bottom panel*). *B*, immunofluorescence showing NF-κB expression (*green signal*) in cells stimulated with 20 ng/ml LPS from *P. aeruginosa* (*center panel*) and treated with 20 μm SET-M33 (*right panel*) as described under “Experimental Procedures.” The control consisted of cells not stimulated with LPS (*left panel*). The membranes were stained with Lectin-Atto 647 (*red*). The green signal was abolished in cells treated with SET-M33.

To determine the inhibitory effect of SET-M33 on production of NF-κB, an essential intracellular factor produced by macrophages in response to stimulation by LPS, protein expression was analyzed in RAW264.7 macrophages after LPS stimulation and treatment with SET-M33. Western blotting with antibodies specific for NF-κB showed that the peptide completely inhibited NF-κB expression in cells stimulated with 25 ng/ml LPS from *K. pneumoniae* (Fig. 2*A*), and immunofluorescence confirmed the disappearance of the NF-κB signal in cells stimulated with 20 ng/ml LPS from *P. aeruginosa* (Fig. 2*B*).

##### Inhibition of Inflammatory Cytokines in Cystic Fibrosis Cells

**FIGURE 3.**
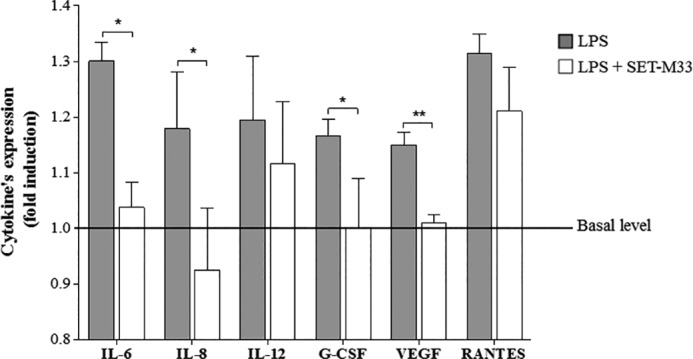
**Bio-plex analysis of cytokines expressed in IB3–1 cells induced with LPS from *P. aeruginosa* and treated with SET-M33.** Data are mean ± S.D. Protein expression is reported in relative expression units (-fold induction) on the *y* axis. IL-6, IL-8, IL-12, and G-CSF were stimulated with 20 ng/ml LPS and treated with 1 μm SET-M33. VEGF was stimulated with 20 ng/ml LPS and treated with 10 μm SET-M33. RANTES was stimulated with 40 ng/ml LPS and treated with 1 μm SET-M33. Basal levels of cytokine expression were as follows: 130.8 pg/ml for IL-6, 114.8 pg/ml for IL-8, 35.8 pg/ml for IL-12, 16.3 pg/ml for G-CSF, 365 pg/ml for VEGF, and 169.7 for RANTES. *, *p* < 0.05; ** *p* < 0.01.

With a view to possible application of SET-M33 in CF lung infections, we analyzed the inhibition of inflammatory cytokines in IB3–1 bronchial cells isolated from a CF patient (20). The effects of SET-M33 on cytokine production in CF cells were evaluated by Luminex technology (21), detecting and quantifying a panel of proteins excreted into the medium. Cells were incubated for 24 h with LPS from *P. aeruginosa* or with LPS and SET-M33 at the concentration indicated in the legend of Fig. 3. Cells treated with SET-M33 showed the following inhibition with respect to cells incubated with LPS alone: IL6 and IL8, > 20%; G-CSF, >14%; VEGF, >12%; IL12, > 6%; Rantes, 8% (Fig. 3).

##### In Vivo Neutralization of LPS

**FIGURE 4.**
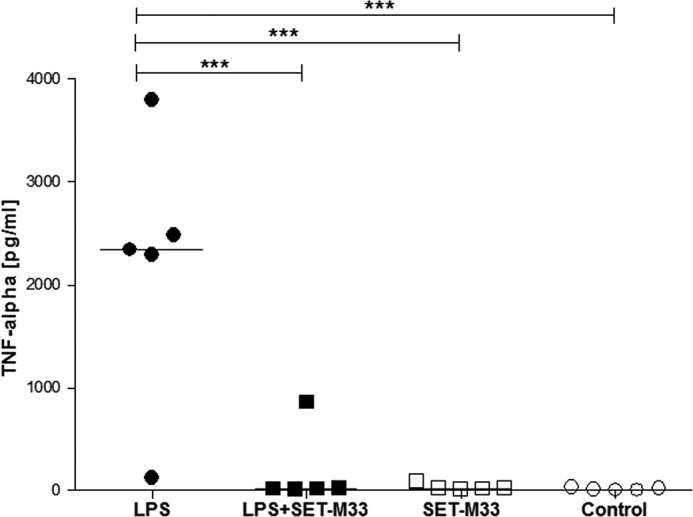
**LPS neutralization *in vivo*.** TNF-α levels (*y* axis) in BAL of mice (each *square* or *circle* indicates one mouse) challenged with *P. aeruginosa* LPS (*black circles*), LPS with SET-M33 (*black squares*), or SET-M33 alone (*white squares*). Controls (*white circles*) show the TNF-α basal level in untreated mice. The *horizontal lines* in the graph indicate median values. ***, *p* < 0.001 compared with the LPS group.

To confirm the LPS neutralization assays obtained *in vitro*, we set up an animal model in which mice were challenged by intratracheal nebulization of LPS from *P. aeruginosa* with or without SET-M33. Nebulization was performed with a Penn Century device following the instructions of the manufacturer. Animals were sacrificed 8 h after the challenge, and bronchoalveolar lavage was collected for TNF-α measurement. Fig. 4 shows that animals treated with LPS and SET-M33 (5 mg/kg) produced about 99% less TNF-α than animals challenged with LPS only. SET-M33 alone did not stimulate any TNF-α production.

##### In Vitro Cell Migration Assay

**FIGURE 5.**
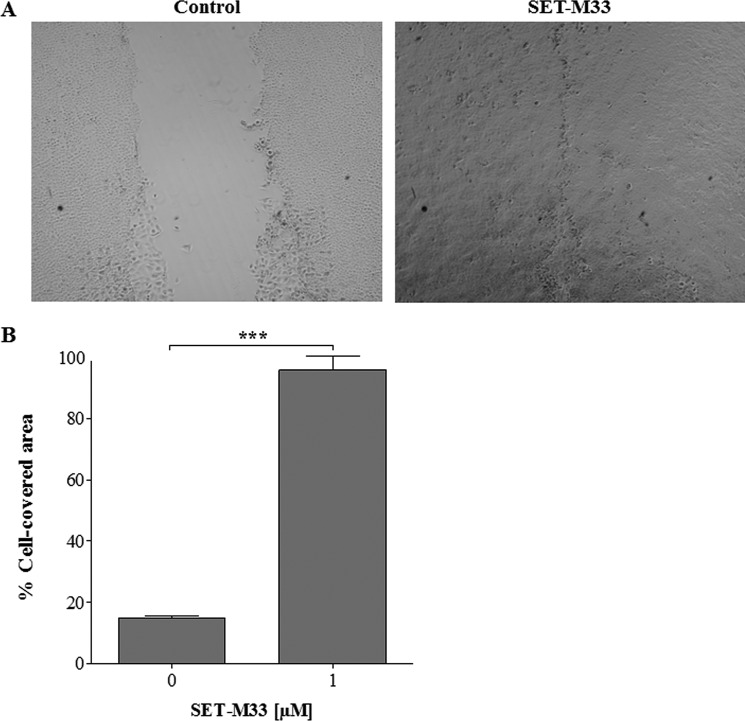
**Effects of SET-M33 peptide on wound closure in a monolayer of HaCaT cells.** *A*, wounds at 24 h photographed by phase contrast light microscopy in control wells (*left panel*) and in wells incubated with SET-M33 1 μm (*right panel*). *B*, histograms of the cell-covered area (percentage) in wells without SET-M33 (*left column*) and with SET-M33 (*right column*) obtained using Tscratch software. All data are the mean ± S.D. of three independent experiments. ***, *p* < 0.0001.

Many natural antimicrobial peptides are known to promote cell migration, thus contributing to the healing of tissue damage such as that caused by *P. aeruginosa* to lung epithelia (22). To evaluate the ability of SET-M33 to promote cell migration, an *in vitro* assay was performed with keratinocytes (HaCaT) cultured in wells where silicon spacers were used to create a gap in the cell monolayer. SET-M33 1 μm promoted closure of the void area within 24 h, whereas only 15% of the void contained cells in untreated cultures (Fig. 5). Gap closure was obtained solely by cell motility and not cell division, as demonstrated by a cell proliferation assay in which cells were incubated with increasing concentrations of SET-M33 (data not shown).

## Discussion

AMPs are considered one of the rare options to use as an alternative or in combination with traditional antibiotics that tend to lead to selection of resistant bacteria. AMPs have a positive net charge that allows them to interact selectively with anionic bacterial membranes and other negatively charged structures, such as LPS. This binding to LPS not only activates a mechanism by which AMPs kill bacteria but, in some cases, also neutralizes LPS, promoting a sort of anti-inflammatory activity. AMPs can therefore be considered simultaneously antibacterial and anti-inflammatory, making them candidate drugs for diseases such as CF in which bacterial infections and inflammation together play a crucial role in disease progression.

SET-M33 has already been reported for its efficacy in killing bacterial pathogens *in vitro* and *in vivo* (10). Here we report its *in vitro* and *in vivo* activity in modulating cell response to inflammatory stimuli (LPS) from different bacteria of clinical interest and its capacity to promote cell migration.

SET-M33 is already known to abate TNF-α production *in vitro* in cells stimulated with LPS from *P. aeruginosa* and *K. pneumoniae* (9), but nothing was published regarding its inhibitory effect on other cytokines and enzymes that together establish inflammatory processes triggered by bacterial infection. We therefore analyzed the effect of SET-M33 on 13 secreted proteins involved in inflammation, including cytokines (TNF-α, IL1-β, IL6, IL8, IL12, MIP1, MIP2, RANTES, KC, IP10, and MCP-1) and growth and stimulating factors (VEGF and G-CSF, respectively), two enzymes responsible for the production of inflammatory mediators (COX-2 and iNOS), and the major intracellular signal transduction agent (NF-κB) involved in the cell response to LPS. All of these inflammatory agents proved to be inhibited by SET-M33 when cells were treated with the peptide after stimulation with LPS from *P. aeruginosa*, *K. pneumoniae*, or *E. coli*. Different experimental techniques, including RT-PCR, Western blotting, immunofluorescence, and Luminex technology, were used, all of them confirming that SET-M33 was able to neutralize LPS.

SET-M33 was also analyzed for its wound repair activity in cultured cells. The results showed that cells treated with SET-M33 promoted wound closure more rapidly than untreated cells.

Lung hyperinflammation, characterized by increased production of various cytokines and agents, including those analyzed in this study, along with a large number of immune cells (23), is recognized as a key factor in CF lung destruction. Inflammation and hyperinflammation of CF lung tissue are principally caused by the basic genetic defect but aggravated by the presence of LPS released by dead bacteria or retained in live bacterial cells. The use of anti-inflammatory drugs such as ibuprofen has proven effective in slowing the progression of lung disease. Unfortunately, the high doses needed to obtain a beneficial outcome are associated with side effects, limiting its use among CF patients (24). There is clearly a need to find new therapies for controlling hyperinflammation in CF.

SET-M33 greatly reduced TNF-α production *in vivo* when LPS from *P. aeruginosa* and the peptide were nebulized directly into mouse lungs. The plan is to administer SET-M33 to CF patients by aerosol. After demonstration of its bactericidal activity *in vivo* by intratracheal inoculation (10), the demonstration of a reduction in major inflammatory cytokines *in vivo* is of great significance for the treatment of CF patients with this molecule.

SET-M33 is currently in preclinical development as a new aerosol drug for resistant lung infections in CF patients. Its potential as a strong LPS inhibitor makes this molecule a candidate for treating the most severe cases of lung damage in CF.

## Experimental Procedures

### Peptide Synthesis

SET-M33 was produced in tetrabranched form by solid-phase synthesis using standard Fmoc (*N*-(9-fluorenyl)methoxycarbonyl) chemistry on Fmoc4-Lys2-Lys-b-Ala Wang resin with a Syro multiple peptide synthesizer (MultiSynTech, Witten, Germany). Side chain-protecting groups were 2,2,4,6,7-pentamethyldihydrobenzofuran-5-sulfonyl for Arg, *t*-butoxycarbonyl for Lys, and *t*-butyl for Ser. The final product was cleaved from the solid support, deprotected by treatment with TFA containing tri-isopropylsilane and water (95/2.5/2.5), and precipitated with diethyl ether. Crude peptide was purified by reverse-phase chromatography on a column for medium-scale preparation in linear gradient form for 30 min using 0.1% TFA/water as eluent A and methanol as eluent B. The purified peptide was obtained as trifluoroacetate salts (TFacetate). Exchange from TFacetate (toxic by-product) to acetate form was carried out using a quaternary ammonium resin in acetate form (AG1-X8, 100–200 mesh, 1.2 meq/ml capacity). The resin-to-peptide ratio was 2000:1. Resin and peptide were stirred for 1 h, the resin was filtered off and washed extensively, and the peptide was recovered and freeze-dried (17). Final peptide purity and identity were confirmed by reverse-phase chromatography on a Phenomenex Jupiter C18 analytical column (300 Å, 5 mm, 25064.6 mm) and by mass spectrometry MALDI-TOF/TOF.

### Cell Cultures

RAW264.7 murine macrophages and HaCaT human keratinocytes, purchased from Istituto Zooprofilattico Sperimentale (Brescia, Italy), were grown in their recommended medium, DMEM supplemented with 10% fetal calf serum, 200 μg/ml glutamine, 100 μg/ml streptomycin, and 60 μg/ml penicillin, and maintained at 37 °C under 5% CO_2_. IB3–1 cells (ATCC), derived from a CF patient with a ΔF508/W1282X mutant genotype and immortalized with adeno12/SV40, were grown in LHC-8 supplemented with 5% FBS in the absence of gentamycin at 37 °C under 5% CO_2_ (20).

### In Vitro Anti-inflammatory Activity

#### 

##### Gene Expression

RAW264.7 cells were seeded in 6-well plates (5 × 10^5^ cells/well) and cultured overnight in a CO_2_ incubator. They were stimulated with 50 ng/ml LPS from *P. aeruginosa* (serotype 10, strain ATCC 27316, Sigma-Aldrich, L 9143), 20 ng/ml LPS from *K. pneumoniae* (strain ATCC 15380, Sigma-Aldrich, L 4268), or 20 ng/ml LPS from *E. coli* (026:B6, Sigma-Aldrich, L 8274) in the presence of 10 or 1 μm SET-M33 in DMEM for 6 h at 37 °C. Total RNA was extracted using an RNeasy kit (Qiagen, Germantown, MD) according to the instructions of the manufacturer. One-step RT-PCR (Qiagen) was applied for retrotranscription and mouse cDNA amplification of IL1-β (330 bp), MIP1 (368 bp), MIP2 (325 bp), TNF-α (795 bp), iNOS (314 bp), IL-6 (474 bp), COX-2 (470 bp), GM-CSF (508 bp), KC (391 bp), IP10 (127 bp), and MCP-1(271 bp). The following oligonucleotides were used as primers. The IL-1β primers were 5′-CTG TCC TGA TGA GAGCAT CC-3′ (sense) and 5′-TGT CCA TTG AGG TGG AGA GC-3′ (antisense). The MIP-1 primers were 5′-ATG AAG CTC TGC GTG TCT GC-3′ (sense) and 5′-TGA GGA GCA AGG ACG CTT CT-3′ (antisense). The MIP-2 primers were 5′-ACA CTT CAG CCT AGC GCC AT-3′ (sense) and 5′-CAG GTC AGT TAG CCT TGC CT-3′ (antisense). The TNF-α primers were 5′-GTT CTG TCC CTT TCA CTC ACT G-3′ (sense) and 5′-GGT AGA GAA TGG ATG AAC ACC-3′ (antisense). The iNOS primers were 5′-CTG CAG CAC TTG GAT CAG GAA CCT G-3′ (sense) and 5′-GGG AGT AGC CTG TGT GCA CCT GGA A-3′ (antisense). The IL-6 primers were 5′-CAT GTT CTC TGG GAA ATC GTG G-3′ (sense) and 5′-AAC GCA CTA GGT TTG CCGA GTA-3′ (antisense). The GM-CSF primers were 5′-TTT CCT GGG CAT TGT GGT CT-3′ (sense) and 5′-AGT TCC TGG CTC ATT ACG CA-3′ (antisense). The COX-2 primers were 5′-TTT GCC CAG CAC TTC ACC CAT-3′ (sense) and 5′-AAG TGG TAA CCG CTC AGG TGT-3′ (antisense). The KC primers were 5′-ACT GCA CCC AAA CCG AAG TCA TAG-3′ (sense) and 5′-GCA CAG TGG TTG ACA CTT AGT GGT-3′ (antisense). The IP-10 primers were 5′-GCC GTC ATT TTC TGC CTC AT-3′ (sense) and 5′-GCT TCC CTA TGG CCC TCA TT-3′ (antisense). The MCP-1 primers were 5′-TTG GCT CAG CCA GAT GCA GTT A-3′ (sense) and 5′-AAC TGC ATC TGC CCT AAG GTC TTC-3′ (antisense).

The following PCR conditions were applied: for IL1-β, MIP2, iNOS, and TNFα, 25 denaturing cycles at 94 °C for 60 s, annealing at 55 °C for 90 s, and extension at 72 °C for 60 s; for MIP1, 20 denaturing cycles at 94 °C for 60 s, annealing at 55 °C for 90 s, and extension at 72 °C for 60 s; for KC and MCP1, 30 denaturing cycles at 94 °C for 60 s, annealing at 54 °C (KC) and 55° (MCP1) for 60 s, and extension at 72 °C for 60 s; for IP10, 25 denaturing cycles at 94 °C for 60 s, annealing at 54 °C for 60 s, and extension at 72 °C for 60 s; for IL-6 and GM-CSF, 30 denaturing cycles at 94 °C for 30 s, annealing at 57 °C for 30 s, and extension at 72 °C for 60 s; and for COX-2, 25 denaturing cycles at 94 °C for 30 s, annealing at 57 °C for 30 s, and extension at 72 °C for 60 s.

##### Western Blotting

RAW264.7 cells were seeded in 6-well plates (3 × 10^6^ cells/well) and cultured overnight in a CO_2_ incubator. Cells were stimulated with 25 ng/ml LPS from *K. pneumoniae* in the presence of 10 μm SET-M33 in DMEM for 6 h at 37 °C. After incubation, cells were washed twice with PBS and detached with ice-cold PBS. They were then centrifuged, suspended in lysis buffer (20 mm Tris-HCl (pH 7.4), 150 mm NaCl, 1% Nonidet P-40, 0.1% SDS, 0.5% cholic acid, and protease inhibitor mixture) and incubated for 30 min on ice. Products of cell lysis were centrifuged at 12,000 rpm for 10 min at 4 °C, and the concentration of proteins in supernatant (cytoplasmic extract) was determined by Bradford assay (Bio-Rad). 20 μg of total proteins was separated by 12% SDS-PAGE and transferred to a nitrocellulose membrane (Bio-Rad). The membrane was blocked in PBS-5% BSA for 1 h at room temperature and then incubated overnight at 4 °C with antibodies specific for NF-κB p65 (Cell Signaling Technology) and for β-actin (Sigma-Aldrich) diluted in PBS-5% BSA-0.1% Tween 20. After washing with PBS-0.05% Tween 20, the membrane was incubated with horseradish peroxidase-conjugated anti-rabbit IgG or anti-mouse IgG (Sigma-Aldrich). Signals were detected using Image LAS4010 (GE Healthcare).

##### Immunofluorescence

RAW264.7 cells were plated at a density of 3 × 10^4^ cells/well in 24-well plates with coverglass slides and then treated with 20 ng/ml LPS *P. aeruginosa* ATCC 27316 serotype 10 (Sigma-Aldrich, L9143) in the presence of 20 μm SET-M33 for 6 h at 37 °C, fixed by incubation with a PBS-4% paraformaldehyde solution for 15 min, saturated for 60 min at room temperature with PBS, 5% normal bovine serum, and 0.3% TritonX-100, and incubated with anti NF-κBp65 rabbit mAb (Cell Signaling Technology) diluted 1:50 in PBS-1% BSA, 0.3% Triton X-100 overnight at 4 °C and then with anti-rabbit-FITC (Sigma-Aldrich) for 1 h at room temperature. Membranes were stained with Lectin Atto-647. NF-κB expression was analyzed by confocal laser microscope (Leica TCS SP5) with 488/520- to 540-nm excitation and 633/660- to 690-nm emission filters for FITC and Atto-647, respectively. All images were processed using ImageJ software (National Institutes of Health).

##### Bio-plex Analysis

SET-M33 (1–10 μm) was incubated for 10 min with LPS (20 and 40 ng/ml) and then added to the IB3–1 cells. After 24 h, the supernatants were separated, centrifuged, and used for the Bio-Plex cytokine assay (Bio-Rad) as described by the manufacturer (Luminex Technology). Briefly, 50 μl of cytokine standards (diluted to 2 μg/μl in culture medium spiked with FBS 5%) or supernatants spiked with BSA 0.5% were incubated with 50 μl of anti-cytokine-conjugated beads in 96-well filter plates for 30 min at room temperature with shaking. Plates were then washed three times by vacuum filtration with 100 μl of Bio-Plex wash buffer. 25 μl of diluted detection antibody was added, and the plates were incubated for 30 min at room temperature with shaking. After three filter washes, 50 μl of streptavidin-phycoerythrin was added, and the plates were incubated for 10 min at room temperature with shaking. Finally, the plates were washed three times by vacuum filtration, the beads were suspended in Bio-Plex assay buffer, and the samples were analyzed on a Bio-Rad 96-well plate reader using the Bio-Plex suspension array system and Bio-Plex Manager software (Bio-Rad). The *p* value was calculated by one-tailed Student's *t* test using GraphPad Prism 5.0 software, and the value is reported in the figure legends.

### In Vivo LPS Neutralization

Animal procedures were approved by the Italian Ministry of Health on September 21, 2012. BALB/c mice (Charles River), 8 weeks old and weighing ∼20 g, were used for the experiment. The animals were maintained and handled in accordance with the Guidelines for Accommodation and Care of Animals (European Convention for the Protection of Vertebrate Animals Used for Experimental and Other Scientific Purposes) and internal guidelines. Prior to experimentation, all mice were allowed at least 4 days of acclimation following shipment.

Mice were anesthetized with Zoletil (0.2 ml/kg) plus Xilor (0.2 ml/kg) and treated with 25 μg/kg LPS from *P. aeruginosa* (serotype 10, strain ATCC 27316, Sigma-Aldrich, L 9143) and 5 mg/kg SET-M33, dissolved in saline solution, through an intratracheal aerosol sprayer (MicroSprayer® IA-1C, Penn Century). Eight hours after intratracheal nebulization, mice were anesthetized and sacrificed by CO_2_ inhalation for collection of bronchoalveolar lavage (BAL). BAL was collected by introduction of 1 ml of saline solution into the lungs via a 22-gauge catheter connected to a syringe. TNF-α concentrations in BAL were assessed by ELISA (ELISA MAX^TM^ Deluxe Sets, BioLegend, San Diego, CA) according to the instructions of the manufacturer.

The data were processed by one-way analysis of variance and Dunnett post tests using GraphPad Prism 5.0 software. The levels of statistical significance are indicated in the figure legends.

### Migration Assay

The peptide ability to stimulate migration of epithelial cells *in vitro* was studied according to a modified scratch assay (25–27). Briefly, HaCaT cells (2 × 10^5^ cells/well) were seeded on each side of a culture insert for live cell analysis (Ibidi, Munich, Germany). Inserts were placed in wells of a 24-well plate and incubated at 37 °C under 5% CO_2_ to allow cells to grow to confluence. Afterward, the inserts were removed with sterile tweezers to create a cell-free area (wound area) of ∼500 μm. The cells were treated with 1 μm SET-M33 peptide in DMEM supplemented with 5% FBS and allowed to migrate in a suitable incubator. At time 0 and after 24 h (T0 and T24), wound areas were visualized under an inverted microscope (Zeiss microscopy) at ×4 magnification and photographed with a Nikon ACT-1 version 2.63 camera. The percentage of cell-covered area at the two times was determined by Tscratch software. The *p* value was calculated by one-tailed Student's *t* test using GraphPad Prism 5.0 software, and the value is reported in the figure legends.

## Author Contributions

J. B. and A. P. coordinated the whole project and wrote the article. C. F. and L. B. synthesized and purified peptides. J. B., G. R., and L. Q. performed *in vitro* experiments for migration and gene expression of inflammatory cytokines and *in vivo* LPS neutralization. I. L. and R. G. performed the Bio-plex analysis.

## Footnotes

3 The abbreviations used are:

AMP: antimicrobial peptide
CF: cystic fibrosis
KC: keratinocyte chemoattractant
Fmoc: *N*-(9-fluorenyl)methoxycarbonyl
BAL: bronchoalveolar lavage
iNOS: inducible nitric oxide synthetase.
